# Depression among HIV/AIDS Sudanese patients: a cross-sectional analytic study

**DOI:** 10.11604/pamj.2017.26.43.10919

**Published:** 2017-01-30

**Authors:** Abdulateef Elbadawi, Hyder Mirghani

**Affiliations:** 1 Community Medicine Department, Faculty of Medicine, University of Tabuk, Tabuk, Saudi Arabia; 2 Medical Department, Faculty of Medicine, University of Tabuk, Tabuk, Saudi Arabia

**Keywords:** HIV/AIDS, depression, Sudan

## Abstract

**Introduction:**

Depression and HIV/AIDS are common morbid health problems; the relationship is bidirectional exacerbating each other with deleterious consequences. There are limited studies on this topic in Sudan. In this study, we investigated depression among HIV/AIDS in Sudan.

**Methods:**

A cross-sectional analytic study was conducted among 362 HIV/AIDS patients from three centers in Khartoum, Sudan. Data were collected by the Hospital Depression and Anxiety (HADS) questionnaire. Chi-square was used for testing the significance and a P. Value of ≥ 0.05 was considered as statistically significant.

**Results:**

Depression was evident in 332 (63.1%) of patients 68 (19.3%) had mild depression, 114 (32.4%) moderate depression, and 40 (11.4%) severe depression. Depression was commoner among women, illiterate, married/widowed, not receiving counseling, delaying the result of the test, P-value <0.05, no significant differences were found regarding test and treatment type P- Value >0.05.

**Conclusion:**

Depression was prevalent among HIV/AIDS patients, especially females, low level of education, and widowed/married patients, and those not receiving counseling and post diagnosis sessions.

## Introduction

HIV/AIDS is a major public health concern with about half of new infections occurring in young people; it has become one of the most devastating diseases humanity has ever faced. Sub-Saharan Africa is one of the most severely affected regions in spite the fact that only 10% of the Word population live in this region, with the highest prevalence among the age 15-24 years, putting more pressure on health authorities and policy makers to take measures to reduce mortality and morbidity among this productive age group [1-3]. Depression is usually missed or dismissed by primary health physicians despite its high prevalence in the community. It is estimated that about one-half to two-thirds are missed in primary care clinics [4]. People with the diagnosis of HIV/AIDS often face social stigma which adds more to their suffering; they have the restriction of employment and marriage, in some cases it may lead to divorce and family rejection [5]. Patients with HIV may feel anxious due to many reasons like fear of disease progression, body decay, pain, and death [6]. AIDS may affect the brain leading to depression as were AIDS medications, on the other hands depression may affect the patient´s adherence to drugs and may enhance the progression of HIV infection towards AIDS. We are not aware of researchers who have studied depression among HIV/AIDS patients in Sudan; thus, we conducted this research to investigate the prevalence of this serious disorder among Sudanese patients with HIV/AIDS.

## Methods

This cross-sectional descriptive study was carried out at Khartoum Hospital during the period from January 2015-January 2016. Three hundred and sixty-two patientswith the diagnosis of HIV were interviewed using the HAD Anxiety and Depression Scale (HADS). The Questionnaire is well validated for use as a screening tool for depression in general medical outpatient clinics [7] but is now widely used in clinical practice and research. This scale consists of seven questions: if still enjoying things, can laugh and see the funny side of things, feel cheerful, if feel as if slowed down, lose of interest in appearance, looking forwards with the enjoyment to things, can enjoy a book, Radio, and TV program, each rating from zero to 3 with zero= nothing and 3= maximum dysfunction. A score of < 7 is regarded as normal, (8-10) mild depression, (11-14) moderate depression and (15-21) severe depression. Data collected include age, sex, education, marital status, place of diagnosis, the probable cause of the disease (Feeling, formal, infected, or marriage), if the patients were counseled or not, type of counseling (couple vs. individual), patients response to the diagnosis of HIV (if accepted or rejected), the time of breaking the news to the patient (immediate or delayed), and the cause of delay if present. All participants signed a written informed consent. Patients were assured that the information collected will be treated confidentially and only for the purpose of this research and that their care will not be affected by any means. Data were analyzed by the Statistical Package for Social Sciences software (SPSS version 20). The chi-square was used for testing the statistical significance; data were presented as percentages or mean ± SD unless otherwise specified and a P-value of less than 0.05 wasconsidered as statistically significant. The ethical committees of Khartoum University and Khartoum Hospital approved the research.

## Results

They were 362 patients with HIV, their ages ranged from 20-40 years, 220 (62.5%) were males, 120 (34.1%) were illiterate, 82 (23.3%) had primary education, 98 (27.85) had secondary education, while 52 (14.8%) were University graduates. One hundred and twenty-two (34.7%) were single, 102 (29%) were married, 94 (26.7%) were divorced, and 32 (9.1%) were widows. Table 1 showed other patients characteristics. In the present study 6 (1.7%) received type 1A treatment, 12 (3.4%) received type 1B treatment, while 36 (10.2%) were on type 1C treatment, other modalities of treatment were shown in Table 2. Table 3 depicted depression among HIV patients in which: Depression was concluded in332 (63.1%) of patients 68 (19.3%) mild depression, 114 (32.4%) moderate depression, and 40 (11.4%) severe depression. In the current study depression was commoner among females (68.2% vs.60%) with significant statistical difference P-value 0.002. A statistically significant difference P-value 0.014 was evident regarding the level of education with the highest rate among illiterate patients (73.3%). The highest rate of depression was reported among married people (71.8%) with significant statistical difference P-value 0.001. Depression was more common among patients who did not receive counseling with high significant statistical difference P< 0.001. Other patient’s characteristics about depression were illustrated in Table 4.

**Table 1:** Basic characteristics of HIV patients

Character	No%
**Sex**	
Males	220 [62.5%]
Females	132 [37.5%]
**Education**	
Illiterate	120 [34.1%]
Primary	82 [23.3%]
Secondary	98 [27.85]
University	52 [14.8%]
**Marital status**	
Single	122 [34.7%]
Married	102 [29%]
Divorced	94 [26.7%]
Widow	32 [9.1%]
**Center type**	
ART	228 [64.8%]
Blood	2 [0.6%]
Mobile	28 [8%]
PMTCT	8 [2.3%]
TB/HIV	30 [8.5%]
VCT	56 [15.9%]
**Diagnosis**	
Volunteer	246 [69.9%]
Referred	106 [30.1%]
**Cause test**	
Exposure	38 [10.8%]
Feeling	208 [59.15]
Formal	42 [11.9%]
Infected	44 [12.5%]
Marriage	20 [5,7%]
**Counseling 1**	292 [83%]
**Counseling 2**	348 [98.9%]
**Session**	
Couple	12 [3.4%]
Individual	340 [96.6%]
**Response**	
Accepted	304 [86.4%]
Rejected	48 [13.6%]
**Result**	
The same day	290 [82.4%]
Delayed	62 [17.6%]

**Table 2:** Treatment type of HIV patients

Treatment type	No%
1 A	6 [1.7%]
1B	12 [3.4%]
1C	36 [10.2%]
1D	210 [59.7%]
Second line	4 [1.1%]
Septrin	84 [23.9%]

**Table 3:** Prevalence of depression among HIV patients

Depression [total]	332 [63.1%]
Mild	68 [19.3%]
Moderate	114 [32.4%]
Severe	40 [11.4%]

**Table 4:** Depression relation to patient’s characteristics

Character	Depression %	P-value
**Sex**		0.002
Males	60	
Females	68.2	
**Education**		0.014
Illiterate	73.3	
Primary	60.9	
Secondary	61.2	
University	46.1	
**Marital status**		0.001
Single	54.1	
Married	71.8	
Divorce	61.7	
Widow	68.7	
**Center type**		0.000
ART	57.8	
Blood bank	0%	
Mobile V	57.1	
PMTCT	100	
TB/HIV	86.6	
VCT	71.4	
**Test type**		0.226
Referred	65.8	
Voluntary	56.6	
**Etiology**		0.003
Exposure	52.6	
Feeling	59.6	
Formal	85.7	
Infected	68.1	
Marriage	60	
**Counseling 1**		0.000
Done	58.9	
Not done	83.3	
**Counseling 2**		0.075
Done	63.7	
Not done	0%	
**Depression session**		0.000
Individual	65.2	
Couple	0%	
**Patient response**		0.002
Accept	60.5	
reject	79.1	
**Delivery of result**		0.040
Same day	60.6	
Delayed	74.1	
**Treatment type**		0.055
A1	66.6	
B1	83.3	
C1	77.2	
D1	58	
Second line	50	
Septrin	64.2	

## Discussion

In the present study the prevalence of depression among HIV/AIDS patients was 63.1%, similar researchers reported a prevalence of 72.9% in HIV patients [8]; other researchers observed that 58.7% of HIV patients were depressed [9]. The sample size and the diagnostic tools used may explain the difference. The prevalence concluded in the current study was higher than that reported by research conducted among elderly Sudanese population and found a prevalence of 47.5% [10]. Our data in collaboration with these studies pointed out that depression is highly frequent among Sudanese HIV patients. In the present study mild depression was found in 19.3% of patients, while 43.8% of patients had moderate to severe depression, similarly researchers from Ethiopia reported mild depression, and moderate to severe depression in 27.3%, and 42% respectively [11]. In the current study women with HIV were more depressed than men (68.2% vs 60%) with significant statistical difference P-value 0.002, similarly Nogueira et al., [12] had reported an increased prevalence of depression among women. The present data found that depression was commoner among the illiterate patients (73.3%), and lowest among University graduates in accordance with the previous researchers [10]. In the present study, married or divorced//widowers were more likely to have depression than single patients, this can be explained by the fact that single patients may not have the pressure of looking after children or feeling guilty about transferring the disease to their children or husbands, also due to religious issues HIV/AIDS is more stigmatizing to married people than singles. Previous literature [13] concluded that depressed HIV patients were six times more likely to miss the medications with the grave consequences on their health. In the present data, depression was lesser in those who received counseling and depression sessions. Furthermore, groups’ sessions can substantially reduce depressive symptoms. Similar to our findings social and community interventions were found to reduce depressive symptoms and improve the quality of life in HIV patients [14]. In the present study no significant statistical difference was evident between different HIV/AIDS modalities in contrast to previous study that concluded higher rates of depression among those taking the combination of efavirenz, lamivudine and stavudine [15].

## Conclusion

In conclusion, depression is prevalent among patients with HIV/AIDS and was commoner among uneducated and married/widowed women. Group sessions could substantially reduce depression/anxiety symptoms.

### What is known about this topic

- Depression is prevalent among HIV/AIDS patients;
- Depression is commoner among women specially among lower educated.

### What this study adds

- The importance of post diagnostic cessions and counseling in alleviating patients symptoms.
- We studied the effects of various HIV/AIDS therapies on rates of depression.
